# Supplementary material for: Tunica intima compensation for reduced stiffness of the tunica media in aging renal arteries as measured with scanning acoustic microscopy
Source: PLoS One. 2020 Nov 4;15(11):e0234759. doi: 10.1371/journal.pone.0234759 (PMC7641345; doi:10.1371/journal.pone.0234759)
Supplement: S1 Table — (DOCX) [file pone.0234759.s001.docx]

**S1 Table.** **Autopsy cases included in this study.**

| Case No | AGE | SEX | Main Diagnosis |
| --- | --- | --- | --- |
| 2196 | 16 | F | DIC |
| 2135 | 21 | M | Cerebral palsy |
| 2148 | 30 | M | Ileus |
| 2157 | 31 | M | Cerebral infarction |
| 2200 | 35 | F | Acute myelocytic leukemia |
| 2138 | 45 | M | Diabetes mellitus (DM), Pneumonia |
| 2160 | 46 | M | Malignant rheumatoid arthritis |
| 2145 | 47 | F | Breast carcinoma |
| 2189 | 50 | M | Acute promyelocytic leukemia |
| 2172 | 50 | M | Hepatocellular carcinoma, Liver cirrhosis |
| 2164 | 51 | M | Malignant lymphoma |
| 2161 | 51 | M | Liver cirrhosis |
| 2165 | 54 | M | Gastric carcinoma |
| 2166 | 56 | F | Malignant melanoma |
| 2151 | 57 | M | Multiple myeloma |
| 2233 | 58 | M | Cholangiocarcinoma |
| 2227 | 58 | M | Malignant lymphoma |
| 2173 | 58 | M | Malignant lymphoma |
| 2180 | 58 | M | Brain tumor |
| 2191 | 59 | M | Progressive systemic sclerosis, Interstitial pneumonia |
| 2193 | 60 | F | Acute myelocytic leukemia |
| 2235 | 60 | M | Liver cirrhosis |
| 2187 | 61 | M | Ulcerative colitis, Sepsis |
| 2214 | 61 | M | Gastric carcinoma, DIC |
| 2150 | 62 | M | Rheumatoid arthritis |
| 2240 | 62 | M | Lung carcinoma, Hepatocellular carcinoma, Liver cirrhosis |
| 2137 | 65 | M | Multiple myeloma, Interstitial pneumonia |
| 2152 | 65 | F | Gastric carcinoma |
| 2194 | 66 | M | Cerebral infarction, DM |
| 2195 | 66 | M | Esophageal carcinoma |
| 2246 | 66 | F | Rheumatoid arthritis, Amyloidosis |
| 2230 | 66 | M | Malignant lymphoma |
| 2231 | 66 | M | Aortic aneurysm |
| 2139 | 67 | M | Acute myocardial infarction |
| 2162 | 67 | M | Esophageal carcinoma |
| 2205 | 67 | F | Glioblastoma |
| 2248 | 67 | M | Multiple myeloma |
| 2184 | 68 | M | Colon carcinoma, Lung carcinoma |
| 2232 | 69 | M | Gastric carcinoma |
| 2156 | 71 | M | Hepatocellular carcinoma, Liver cirrhosis |
| 2207 | 71 | M | Cholangiocarcinoma, Bladder carcinoma |
| 2238 | 71 | M | Esophageal carcinoma, Pulmonary fibrosis |
| 2215 | 72 | M | Rheumatoid arthritis, Interstitial pneumonia |
| 2219 | 72 | M | Aortic aneurysm |
| 2169 | 73 | F | Breast carcinoma |
| 2158 | 74 | F | Pneumonia |
| 2229 | 74 | M | Gastric carcinoma |
| 2242 | 75 | F | Acute myelocytic leukemia |
| 2204 | 76 | F | Cholangiocarcinoma |
| 2206 | 76 | M | Hepatocellular carcinoma |
| 2213 | 76 | M | Rheumatoid arthritis |
| 2190 | 77 | M | Hepatocellular carcinoma, Liver cirrhosis |
| 2228 | 78 | F | Interstitial pneumonia |
| 2153 | 78 | M | Esophageal carcinoma |
| 2167 | 78 | M | Bullous pemphigoid |
| 2159 | 79 | M | Cerebral infarction |
| 2163 | 80 | M | Acute myocardial infarction |
| 2226 | 81 | M | Malignant lymphoma |
| 2171 | 81 | F | Gastric carcinoma |
| 2182 | 83 | M | Pancreas carcinoma, Cerebral infarction |
| 2245 | 84 | M | Gastric carcinoma |
| 2131 | 85 | F | AL amyloidosis |
| 2155 | 101 | F | Lip carcinoma |

M=63, F=16
