# Supplementary material for: Tunica intima compensation for reduced stiffness of the tunica media in aging renal arteries as measured with scanning acoustic microscopy
Source: PLoS One. 2020 Nov 4;15(11):e0234759. doi: 10.1371/journal.pone.0234759 (PMC7641345; doi:10.1371/journal.pone.0234759)
Supplement: S2 Table — (DOCX) [file pone.0234759.s002.docx]

**S2 Table. Changes in SOS among fresh-frozen samples in different fixatives and FFPE sections. Mean values ± SD were shown.**

ET20s: ethanol 20 seconds, Form3m: formalin 3min, Inner Med: inner medial layer, Outer Med: outer medial layer
