# Supplementary material for: Tunica intima compensation for reduced stiffness of the tunica media in aging renal arteries as measured with scanning acoustic microscopy
Source: PLoS One. 2020 Nov 4;15(11):e0234759. doi: 10.1371/journal.pone.0234759 (PMC7641345; doi:10.1371/journal.pone.0234759)
Supplement: S3 Table — (DOCX) [file pone.0234759.s003.docx]

**S3 Table. Relationship between age and speed-of-sound（SOS of the medial layer of the renal artery).**

|  | AGE (y) | Mean SOS (m/s) |
| --- | --- | --- |
|  | 16 | 1641.9 |
|  | 21 | 1639.8 |
|  | 30 | 1631.7 |
|  | 30 | 1635.8 |
|  | 31 | 1623.2 |
|  | 31 | 1673.6 |
|  | 35 | 1631.5 |
|  | 45 | 1620.9 |
|  | 46 | 1643.7 |
|  | 47 | 1612.4 |
|  | 50 | 1599.5 |
|  | 50 | 1630.1 |
|  | 51 | 1625.2 |
|  | 51 | 1612.8 |
|  | 54 | 1621.6 |
|  | 56 | 1636.0 |
|  | 57 | 1657.3 |
|  | 58 | 1638.2 |
|  | 58 | 1648.4 |
|  | 58 | 1614.5 |
|  | 58 | 1659.1 |
|  | 59 | 1565.3 |
|  | 60 | 1623.4 |
|  | 60 | 1614.5 |
|  | 61 | 1616.7 |
|  | 61 | 1576.2 |
|  | 62 | 1595.3 |
|  | 62 | 1635.2 |
|  | 65 | 1613.9 |
|  | 65 | 1630.4 |
|  | 66 | 1612.2 |
|  | 66 | 1599.2 |
|  | 66 | 1620.8 |
|  | 66 | 1638.9 |
|  | 66 | 1637.8 |
|  | 67 | 1635.0 |
|  | 67 | 1558.1 |
|  | 67 | 1616.0 |
|  | 67 | 1568.5 |
|  | 68 | 1605.0 |
|  | 69 | 1637.2 |
|  | 71 | 1609.1 |
|  | 71 | 1542.6 |
|  | 71 | 1605.3 |
|  | 72 | 1600.4 |
|  | 72 | 1651.5 |
|  | 73 | 1581.1 |
|  | 74 | 1596.7 |
|  | 74 | 1579.4 |
|  | 75 | 1604.5 |
|  | 76 | 1595.4 |
|  | 76 | 1600.2 |
|  | 76 | 1603.0 |
|  | 77 | 1545.2 |
|  | 78 | 1623.1 |
|  | 78 | 1601.8 |
|  | 78 | 1557.5 |
|  | 79 | 1601.8 |
|  | 80 | 1634.5 |
|  | 81 | 1589.5 |
|  | 81 | 1612.8 |
|  | 83 | 1628.8 |
|  | 84 | 1635.5 |
|  | 85 | 1632.2 |
|  | 101 | 1608.4 |
| Mean | 62.91 | 1614.4 |
