# Supplementary material for: Tunica intima compensation for reduced stiffness of the tunica media in aging renal arteries as measured with scanning acoustic microscopy
Source: PLoS One. 2020 Nov 4;15(11):e0234759. doi: 10.1371/journal.pone.0234759 (PMC7641345; doi:10.1371/journal.pone.0234759)
Supplement: S4 Table — (DOCX) [file pone.0234759.s004.docx]

**S4 Table.** **Age-related speed-of-sound values of the internal (IEL) and external (EEL) elastic laminae.**

| Age(y) | IEL SOS (m/s) | EEL SOS (m/s) |
| --- | --- | --- |
| 16 | 1754.8 | 1826.0 |
| 21 | 1736.7 | 1758.3 |
| 30 | 1666.6 | 1756.4 |
| 31 | 1708.6 | 1745.6 |
| 35 | 1676.1 | 1733.5 |
| 45 | 1665.0 | 1740.2 |
| 46 | 1650.3 | 1781.4 |
| 47 | 1680.3 | 1705.5 |
| 50 | 1661.5 | 1725.3 |
| 50 | 1736.3 | 1731.0 |
| 51 | 1723.1 | 1696.1 |
| 51 | 1664.7 | 1750.1 |
| 58 | 1645.2 | 1694.0 |
| 58 | 1678.7 | 1710.3 |
| 60 | 1733.8 | 1794.7 |
| 61 | 1681.0 | 1750.0 |
| 62 | 1647.4 | 1650.2 |
| 65 | 1684.6 | 1728.3 |
| 65 | 1679.7 | 1686.1 |
| 66 | 1644.4 | 1786.3 |
| 66 | 1652.1 | 1696.1 |
| 66 | 1698.5 | 1715.9 |
| 67 | 1685.7 | 1698.8 |
| 76 | 1685.2 | 1708.4 |
| 76 | 1699.3 | 1674.7 |
| 76 | 1686.9 | 1684.8 |
| 78 | 1648.8 | 1713.9 |
| 78 | 1679.5 | 1685.6 |
| 79 | 1690.7 | 1730.1 |
| 80 | 1664.8 | 1696.3 |
| 81 | 1750.3 | 1718.1 |
| 81 | 1637.0 | 1675.1 |
| 83 | 1636.1 | 1704.6 |
| 84 | 1701.4 | 1645.7 |
| 85 | 1658.9 | 1690.9 |
| 101 | 1672.9 | 1660.6 |
| Mean | 1682.4 | 1718.0 |
| SD | 31.789 | 40.35 |

N=36
