# Supplementary material for: Tunica intima compensation for reduced stiffness of the tunica media in aging renal arteries as measured with scanning acoustic microscopy
Source: PLoS One. 2020 Nov 4;15(11):e0234759. doi: 10.1371/journal.pone.0234759 (PMC7641345; doi:10.1371/journal.pone.0234759)
Supplement: S5 Table — (DOCX) [file pone.0234759.s005.docx]

**S5 Table. Mean blood pressure and speed-of-sound values for the tunica media of the renal artery.**

| Age (y) | Mean BP (mmHg) | Mean SOS (m/s) |
| --- | --- | --- |
| 16 | 60.0 | 1641.9 |
| 21 | 73.3 | 1639.8 |
| 30 | 75.7 | 1633.8 |
| 31 | 76.3 | 1648.4 |
| 45 | 98.0 | 1620.9 |
| 46 | 93.3 | 1643.7 |
| 51 | 79.3 | 1612.8 |
| 51 | 90.7 | 1625.2 |
| 56 | 90.7 | 1636.0 |
| 57 | 96.0 | 1657.3 |
| 58 | 78.3 | 1648.4 |
| 58 | 85.7 | 1638.2 |
| 58 | 84.3 | 1614.5 |
| 60 | 137.3 | 1614.5 |
| 61 | 107.3 | 1576.2 |
| 62 | 110.7 | 1595.3 |
| 62 | 95.7 | 1635.2 |
| 65 | 76.7 | 1613.9 |
| 66 | 82.0 | 1599.2 |
| 66 | 90.0 | 1620.8 |
| 66 | 93.3 | 1638.9 |
| 67 | 103.3 | 1635.0 |
| 67 | 102.7 | 1558.1 |
| 69 | 86.0 | 1637.2 |
| 71 | 100.0 | 1609.1 |
| 71 | 80.7 | 1542.6 |
| 71 | 98.0 | 1605.3 |
| 74 | 80.0 | 1596.7 |
| 76 | 98.0 | 1600.2 |
| 76 | 104.0 | 1603.0 |
| 78 | 96.7 | 1601.8 |
| 78 | 101.7 | 1623.1 |
| 78 | 130.7 | 1557.5 |
| 81 | 102.0 | 1612.8 |
| 83 | 96.7 | 1628.8 |
| 84 | 94.7 | 1635.5 |
| 85 | 80.7 | 1632.2 |
| Mean | 92.7 | 1617.1 |
| SD | 14.96 | 26.51 |
