# Supplementary material for: Tunica intima compensation for reduced stiffness of the tunica media in aging renal arteries as measured with scanning acoustic microscopy
Source: PLoS One. 2020 Nov 4;15(11):e0234759. doi: 10.1371/journal.pone.0234759 (PMC7641345; doi:10.1371/journal.pone.0234759)
Supplement: S6 Table — (DOCX) [file pone.0234759.s006.docx]

**S6 Table. Association between systolic (SBP) and diastolic (DBP) blood pressure and age.**

| Age (y) | SBP (mmHg) | DBP (mmHg) |
| --- | --- | --- |
| 16 | 80 | 50 |
| 21 | 100 | 60 |
| 30 | 115 | 56 |
| 31 | 111 | 59 |
| 45 | 126 | 84 |
| 46 | 120 | 80 |
| 51 | 110 | 64 |
| 51 | 120 | 76 |
| 56 | 112 | 80 |
| 58 | 115 | 60 |
| 58 | 105 | 76 |
| 58 | 107 | 73 |
| 60 | 196 | 108 |
| 61 | 154 | 84 |
| 62 | 140 | 96 |
| 62 | 129 | 79 |
| 65 | 110 | 60 |
| 66 | 114 | 66 |
| 66 | 130 | 70 |
| 66 | 124 | 78 |
| 67 | 150 | 80 |
| 67 | 128 | 90 |
| 69 | 110 | 74 |
| 71 | 118 | 62 |
| 71 | 140 | 77 |
| 74 | 120 | 60 |
| 76 | 89 | 51 |
| 76 | 150 | 72 |
| 76 | 152 | 80 |
| 78 | 130 | 80 |
| 78 | 153 | 76 |
| 78 | 160 | 116 |
| 81 | 146 | 80 |
| 83 | 130 | 80 |
| 84 | 148 | 68 |
| 85 | 106 | 68 |
| Mean | 126.3 | 74.3 |
| SD | 22.6 | 14.2 |
