# Supplementary material for: Tunica intima compensation for reduced stiffness of the tunica media in aging renal arteries as measured with scanning acoustic microscopy
Source: PLoS One. 2020 Nov 4;15(11):e0234759. doi: 10.1371/journal.pone.0234759 (PMC7641345; doi:10.1371/journal.pone.0234759)
Supplement: S7 Table — (DOCX) [file pone.0234759.s007.docx]

**S7 Table.** **Age-related changes in thickness of each layer of the renal artery.**

| Age | Medial thickness (µm) | Adventitial thickness (µm) | Intimal thickness (µm) |
| --- | --- | --- | --- |
| 16 | 337.8 | 312.3 | 0 |
| 21 | 326.8 | 320.9 | 25.2 |
| 30 | 957.6 | 968.2 | 44.8 |
| 31 | 340.9 | 202.9 | 0 |
| 35 | 878.7 | 1410.9 | 0 |
| 45 | 1047.2 | 635.1 | 124.4 |
| 46 | 357.6 | 260.8 | 56 |
| 47 | 584.9 | 694.3 | 273.7 |
| 50 | 783.3 | 537.8 | 58.3 |
| 51 | 637.6 | 744.1 | 279.7 |
| 51 | 494.1 | 215.1 | 143.2 |
| 58 | 1049 | 639.6 | 300.6 |
| 58 | 396 | 440.6 | 18.7 |
| 60 | 864.2 | 867.7 | 39.3 |
| 61 | 831.6 | 711.1 | 455.1 |
| 62 | 549.2 | 791.9 | 541.8 |
| 65 | 690.2 | 476 | 338.7 |
| 65 | 847.1 | 785.4 | 136.9 |
| 66 | 579.9 | 610.2 | 996.4 |
| 66 | 654.9 | 664.7 | 461.4 |
| 66 | 536.4 | 363.4 | 270.8 |
| 67 | 826.7 | 534.7 | 590.6 |
| 76 | 584.1 | 627.4 | 292.5 |
| 76 | 949.9 | 1495.5 | 599.2 |
| 76 | 686.3 | 756 | 296.5 |
| 78 | 462 | 827.3 | 102.8 |
| 78 | 639.2 | 536.8 | 310.1 |
| 79 | 794.8 | 855.1 | 343.9 |
| 80 | 654.6 | 623.7 | 587.8 |
| 81 | 1175.8 | 868.6 | 384.6 |
| 81 | 833.7 | 772.6 | 650.4 |
| 83 | 658.3 | 894.9 | 215.8 |
| 84 | 1001.3 | 787.4 | 500.2 |
| 85 | 738.4 | 822.6 | 533.5 |
| 101 | 871.6 | 846.9 | 409.1 |
| Mean | 703.5 | 682.9 | 297 |
| SD | 222.2 | 282.5 | 236.1 |

N=35
