# Supplementary material for: Tunica intima compensation for reduced stiffness of the tunica media in aging renal arteries as measured with scanning acoustic microscopy
Source: PLoS One. 2020 Nov 4;15(11):e0234759. doi: 10.1371/journal.pone.0234759 (PMC7641345; doi:10.1371/journal.pone.0234759)
Supplement: S8 Table — (DOCX) [file pone.0234759.s008.docx]

**S8 Table. Age-related changes in the length of the medial inner and outer axes**

| Age (y) | Medial outer axis (µm) | Medial inner axis (µm) |
| --- | --- | --- |
| 16 | 1248.6 | 1183.6 |
| 21 | 2659 | 1149.8 |
| 30 | 3099.7 | 2561.1 |
| 31 | 4726.5 | 1127.5 |
| 35 | 3418 | 2119.5 |
| 45 | 5836.1 | 4308.5 |
| 46 | 4927.2 | 1987.2 |
| 47 | 3164.1 | 2113.2 |
| 50 | 6505.7 | 2346.5 |
| 51 | 3799 | 1693.3 |
| 51 | 3893.4 | 2576.1 |
| 58 | 4676.2 | 2290.6 |
| 58 | 2727.3 | 1733.6 |
| 60 | 2687.6 | 1981.8 |
| 61 | 3491.6 | 3353.3 |
| 62 | 4527.8 | 2592.7 |
| 65 | 6052.7 | 2896 |
| 65 | 3336.8 | 2460.5 |
| 66 | 5661.1 | 3057.3 |
| 66 | 6014.8 | 3034.2 |
| 66 | 3523.1 | 1487.8 |
| 67 | 5301.3 | 2928.5 |
| 76 | 7155.6 | 1736 |
| 76 | 4156.7 | 3506.1 |
| 76 | 3879.5 | 2414.3 |
| 78 | 6543.2 | 1518.2 |
| 78 | 2031.8 | 1767.3 |
| 79 | 4842.1 | 3928.7 |
| 80 | 3578.5 | 3036.5 |
| 81 | 5530.2 | 3567.7 |
| 81 | 3129.1 | 2514.4 |
| 83 | 5323 | 5081.3 |
| 84 | 4178.2 | 3364.8 |
| 85 | 6230.1 | 4999.9 |
| 101 | 4545.6 | 4588.6 |
| Mean | 4354.3 | 2657.3 |
| SD | 1404.0 | 1047.1 |

N=35
