# Supplementary material for: Tunica intima compensation for reduced stiffness of the tunica media in aging renal arteries as measured with scanning acoustic microscopy
Source: PLoS One. 2020 Nov 4;15(11):e0234759. doi: 10.1371/journal.pone.0234759 (PMC7641345; doi:10.1371/journal.pone.0234759)
Supplement: S9 Table — (DOCX) [file pone.0234759.s009.docx]

**S9 Table. Average speed of sound of the renal and aortic medial layer.**

|  | Age (y) | RA medial SOS(m/s) | Ao medial SOS (m/s) |
| --- | --- | --- | --- |
|  | 16 | 1641.9 | 1689.9 |
|  | 21 | 1639.8 | 1693.4 |
|  | 30 | 1633.8 | 1649.6 |
|  | 31 | 1648.4 | 1724.1 |
|  | 35 | 1631.5 | 1677.1 |
|  | 45 | 1620.9 | 1690.2 |
|  | 46 | 1643.7 | 1642.7 |
|  | 47 | 1612.4 | 1713.2 |
|  | 50 | 1630.1 | 1656.4 |
|  | 50 | 1599.5 | 1644.9 |
|  | 51 | 1612.8 | na |
|  | 51 | 1625.2 | na |
|  | 58 | 1648.4 | 1671.4 |
|  | 58 | 1638.2 | 1708.4 |
|  | 60 | 1623.4 | 1645.5 |
|  | 61 | 1616.7 | 1646.7 |
|  | 62 | 1595.3 | 1692.9 |
|  | 65 | 1613.9 | 1665.2 |
|  | 65 | 1630.4 | 1637.7 |
|  | 66 | 1612.2 | 1610.2 |
|  | 66 | 1599.2 | 1612.8 |
|  | 66 | 1620.8 | 1694.2 |
|  | 67 | 1635.0 | 1668.8 |
|  | 76 | 1595.4 | 1620.5 |
|  | 76 | 1600.2 | 1659.5 |
|  | 76 | 1603.0 | 1681.1 |
|  | 78 | 1601.8 | na |
|  | 78 | 1623.1 | 1678.2 |
|  | 79 | 1601.8 | 1658.6 |
|  | 80 | 1634.5 | 1682.2 |
|  | 81 | 1612.8 | 1648.2 |
|  | 81 | 1589.5 | 1577.7 |
|  | 83 | 1628.8 | 1658.7 |
|  | 84 | 1635.5 | 1653.3 |
|  | 85 | 1632.2 | 1648.4 |
|  | 101 | 1608.4 | 1668.7 |
| Mean | 61.81 | 1620.6 | 1662.7 |
| SD | 19.53 | 16.55 | 31.11 |

RA: renal artery, Ao: aorta, na: not available
