# Supplementary material for: Tunica intima compensation for reduced stiffness of the tunica media in aging renal arteries as measured with scanning acoustic microscopy
Source: PLoS One. 2020 Nov 4;15(11):e0234759. doi: 10.1371/journal.pone.0234759 (PMC7641345; doi:10.1371/journal.pone.0234759)
Supplement: S10 Table — (DOCX) [file pone.0234759.s010.docx]

**S10 Table. SOS alteration after collagenase.**

| Case | SOS mean | SD |
| --- | --- | --- |
| Y-0h (n=8) | 1725.1 | 32.7 |
| Y-1.5h (n=7) | 1679.9 | 20.2 |
| Y-3h (n=7) | 1666.5 | 23.5 |
| O-0h (n=6) | 1657.4 | 58.6 |
| O-1.5h (n=6) | 1613.5 | 26.7 |
| O-3h (n=6) | 1592.8 | 57.9 |

Y: young RA, O: old RA
