# Supplementary material for: Tunica intima compensation for reduced stiffness of the tunica media in aging renal arteries as measured with scanning acoustic microscopy
Source: PLoS One. 2020 Nov 4;15(11):e0234759. doi: 10.1371/journal.pone.0234759 (PMC7641345; doi:10.1371/journal.pone.0234759)
Supplement: S1 File — (DOCX) [file pone.0234759.s011.docx]

**S1 File**

**Changes in SOS images of mouse artery among fresh-frozen sections in different fixatives and formalin-fixed paraffin-embedded (FFPE) sections.**

**Materials and Methods**

Fresh mouse arteries were frozen and sectioned with a cryotome. The sections were dried, fixed in 95% ethanol, and then soaked in 10% buffered formalin. The same section was used to compare the images after different fixation conditions. The residual frozen tissue cut with the cryotome were fixed in 10% buffered formalin for one day, embedded in paraffin, and sectioned with a microtome. The deparaffinized and frozen sections were soaked in distilled water and measured with SAM to compare their images.

**Results**

SOS images after 95% ethanol or 10% formalin fixation at different time points showed no remarkable changes (Figs. A and B, Table C). However, FFPE sections significantly increased SOS values on the arterial walls of the smooth muscle layer (P = 8.6E – 09)

**Fig A. SOS images among fresh samples in different fixatives and formalin-fixed paraffin-embedded samples (FFPE).** SOS of fresh-frozen section with different fixatives showed no remarkable changes. However, FFPE sections significantly increased SOS values on the arterial walls of the smooth muscle. The corresponding LM images in HE stain were shown at the same magnification.

Sample A


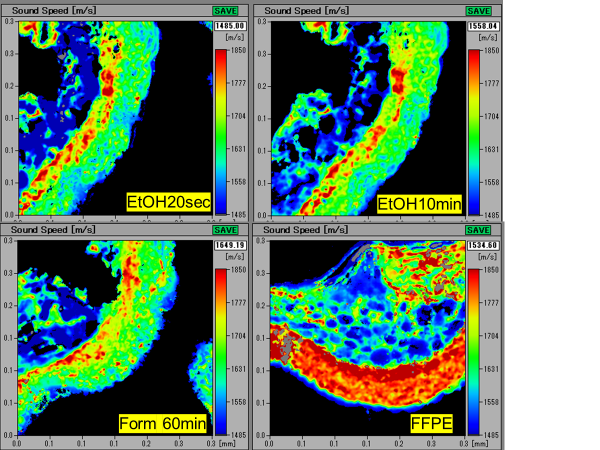


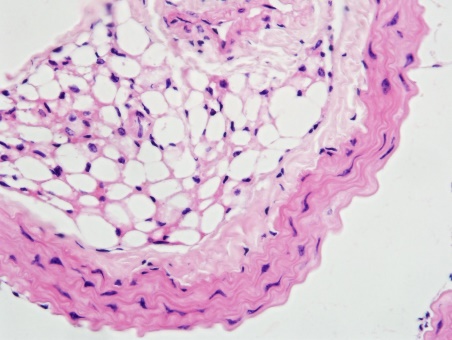


Sample B


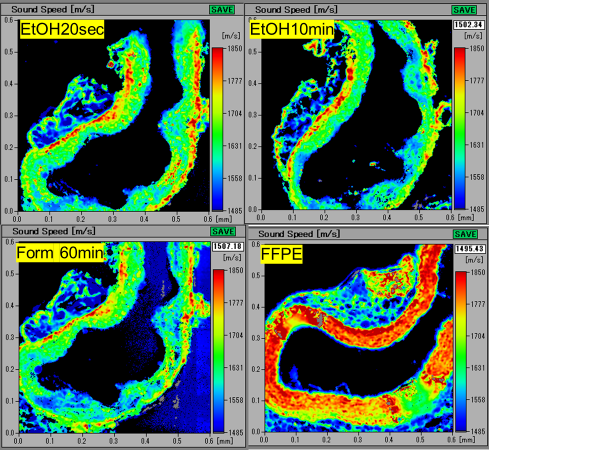
.
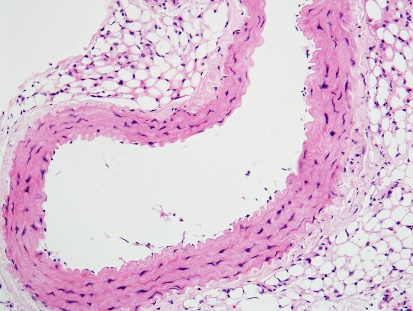


Sample C


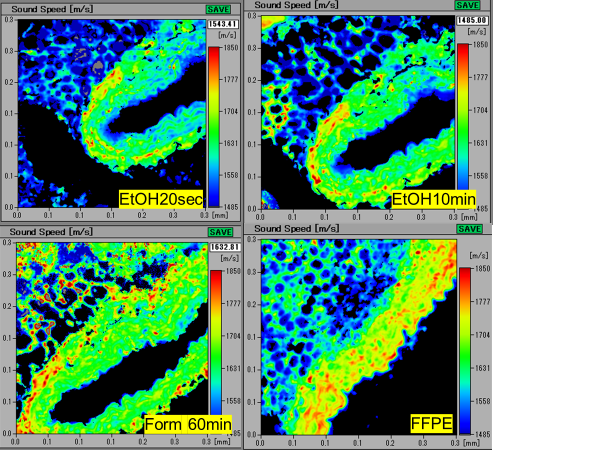


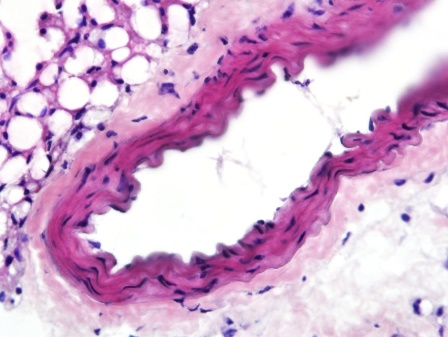


Form: formalin; FFPE: formalin-fixed paraffin-embedded; A, B, C: Different samples

**Fig. B. Comparison of SOS values in different fixatives**

**Table C. SOS values (mean ± SD) among fresh samples in different fixatives and formalin-fixed paraffin-embedded samples.**

|  | EtOH20sec | Et OH10m |  | Form3m | Form60m | FFPE |
| --- | --- | --- | --- | --- | --- | --- |
| A | 1643.1±31.1 | 1628.5±29.4 |  | 1664.9±23.1 | 1674.4±43.8 | 1799.4±17.5 |
| B | 1643.7±30.5 | 1649.0±22.2 |  | 1646.3±23.1 | 1652.9±22.4 | 1781.5±19.7 |
| C | 1625.0±27.9 | 1636.6±13.8 |  | 1653.9±8.4 | 1663.8±17.0 | 1719.5±22.0 |

Form: formalin; FFPE: formalin-fixed paraffin-embedded; A, B, C: Different samples

.
